# Lithium: one drug, five complications

**DOI:** 10.1186/s40560-017-0257-5

**Published:** 2017-12-20

**Authors:** Jeremy Zaworski, Pierre-Yves Delannoy, Nicolas Boussekey, Damien Thellier, Hugues Georges, Olivier Leroy

**Affiliations:** Pierre-Yves Delannoy, Intensive Care Unit, Hôpital Chatiliez, 155 rue du Président Coty, BP 619, 59208 Tourcoing cedex, France

**Keywords:** Lithium, Poisoning, Pancytopenia, Polyneuropathy, Diabetes insipidus

## Abstract

**Background:**

Lithium poisoning could trigger multiple complications. We report the case of a lithium poisoning with five complications that are described for the first time together.

**Case report:**

A 60-year-old woman was admitted in our intensive care unit for altered consciousness. Severe lithium intoxication was diagnosed (lithium plasmatic level 8.21 mmol/l) associated with acute oliguric kidney failure. Continuous renal replacement therapy was started immediately. Orotracheal intubation was quickly required because of status epilepticus. Medullary aplasia happened 48 h after the patient was intubated. Infectious and immunological causes were ruled out and lithium poisoning was considered as the most likely etiology. Iterative blood and platelet transfusion were required. Severe polyneuropathy was diagnosed on the 5th day after admission. The patient showed a peripheral tetraparesia and cranial nerve failure while lithium plasmatic level had decreased to a therapeutic level. Conversely, urine output increased and hypernatremia promptly occurred, which led to diabetes insipidus diagnosis. Neuropathy decreased in 72 h and the patient was definitely extubated by the 11th day. Hematologic disturbances decreased and no blood transfusion would be required after the 8th day. The patient would keep sequellas of the poisoning. Thin motricity would still be altered and polyuria would remain. Diffuse alopecia was promptly observed, with no iron deficiency or thyroid disturbance.

**Conclusion:**

In addition to presenting this case report, we herein discuss the drug causality, the consequences, and the plausible pathophysiology of these five situations.

## Background

Lithium is the first-line treatment for bipolar disorder. Lithium poisoning was historically associated with a 25% mortality [1]. A more recent survey showed that mortality is less than 1% [2], namely thanks to intensive care progress and the more wide-spread use of extracorporeal treatment [2]. Most of the lithium poisonings are associated with infections or drug association [3], the most frequent being with diuretics or angiotensin-converting enzyme inhibitors. Self-administration of toxic doses may account for 20% of the hospitalized poisoning [4].The case we report shows multiple lithium complications: pancytopenia, polyneuropathy, diabetes insipidus nephrogenic, seizure, and alopecia. Those complications are described for the first time together in our case report which illustrates how wide the spectrum of lithium-poisoning-related complications could be.

## Case presentation

A 60-year-old woman was admitted to our intensive care unit (ICU) for altered consciousness. She was receiving lithium for a bipolar disorder for more than 10 years. Her last lithium level was on the therapeutic window (1.19 mmol/l 2 months before the admission) with a 400-mg twice-daily lithium carbonate intake without recent change in her drug regime. Her kidney function was normal (creatinine level 50 μmol/l).

The physical exam showed an altered consciousness (Glasgow score 9) with a bilateral nystagmus. She was apyretic and there was no hemodynamic or respiratory failure. Urine output was less than 100 ml over 6 h.

Her biological exams were as follows: white blood cell 28,640/mm^3^ (85% neutrophils); hemoglobin 15.9 g/dl; platelet count 140,000/mm^3^; prothrombin time 100%; activated partial thromboplastin time 27/32 s; creatinine 184 μmol/l; urea 18 mmol/l; K^+^ 2.9 meq/l; Na^+^ 138 meq/l; Cl^−^ 104 meq/l; blood gaz sample pH 7, 49; PO_2_ 95 mmHg; PCO_2_ 31 mmHg; HCO_3_^−^ 24 meq/l.

ALAT 30 IU/l; ASAT 30 IU/l bilirubin 7 mg/l; screening test for benzodiazepine, barbituric and tricyclic antidepressant were negative. Brain computed tomography did not show any bleeding or ischemic stroke.

The lumbar puncture did not show any sign of cerebral nervous system infection (0 cell/mm^3^; protein level 0.52 g/l; glucose level 0.97 g/l).

The electrocardiogram was normal with a normal QT and PR space and without any pattern of ischemia.

The patient condition deteriorated dramatically after the sixth hour. Status epilepticus happened and orotracheal intubation was required after clonazepam and phenytoin’s failure. Urine output was still under 100 ml over 6 h. Seizure cessation was obtained with midazolam and sufentanil. Antiepileptic therapeutic were settled (levetiracetam and clobazam).

The lithium level was belatedly obtained soon after the patient was intubated. It was at 8.21 mmol/l (therapeutic window 0.7–1.2 mmol/l).

**Fig. 1:**
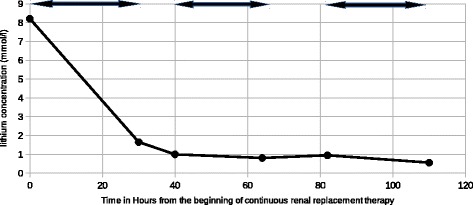
Lithium concentration versus time. Continuous veno-venous haemodiafiltration session is represented with a double *arrow* (↔)

**Fig. 2:**
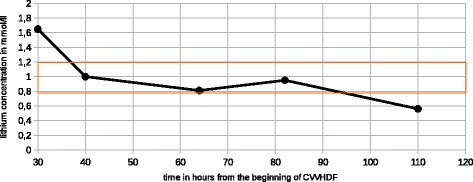
Lithium concentration versus time, from Fig. 1

We decided to begin a continuous extrarenal epuration by veno-venous hemodiafiltration (CVVHDF), the only technic available in our center. Our CVVHDF was obtained thanks to femoral venous access. The mean session duration was 24 h. Mean flow blood rate was 180 ml/min, predilution 600 ml/h, postdilution 1200 ml/h; ultrafiltration rate adapted to volemia estimation. Anticoagulation was obtained with unfractionated heparin. Lithium concentration was determined on admission, at the beginning, and at the end of each CVVHDF session. Lithium level quickly decreased (Fig. 1; Table 1); a rebound happened but it was still on the therapeutic window (Fig. 2).

**Table 1:** Lithium concentration versus time

Time (hours)	Lithium concentration (mmo1/1)
0	8.21
30	1.65
40	1.00
64	0.81
82	0.95
110	0.56

CVVHDF was pursued until the fifth day after admission due to persistent oliguric kidney failure.

Conversely, we noticed a profound bicytopenia: hemoglobin content fell below 8 g/dl by the sixth day and platelets started to fall below 30,000/mm^3^. Hemolysis parameters were normal. Reticulocyte count was low (20,000/mm^3^). The bone marrow aspiration showed a poor bone marrow without any malignancy. Biological exams ruled out any infections (parvovirus B19, hepatitis, HIV), vitamin deficiency, or immunological etiology (antinuclear antibody; anti-neutrophil cytoplasmic antibody). Iterative blood and platelet transfusion was required.

Sedations were taken out by the seventh day. Consciousness was rapidly normal without any seizure happening. We noticed a tetraplegia with depressed reflex: the muscular testing was of 2/5 over each muscular limb. Deglutition and cough was severely altered. The electromyogram showed severe axonal polyneuropathy without demyelinating pattern.

Kidney function quickly went back to normal with a urine output of 1200 ml/24 h and a plasmatic creatinine at 50 μmol/l on day 8. The patient soon became polyuric and the sodium level rose to 146 mmol/l with inappropriate urinary osmolarity (212 mosmol/l) indicating diabetes insipidus. Normal sodium level was obtained with 3500 ml water intake for 24 h.

We noticed diffuse severe alopecia without any scalp lesion.

Muscular weakness rapidly resolved: the muscular testing was mildly altered on the four limbs, gag reflex was efficient and the patient was then able to cough. Extubation was obtained on day 11 without any ventilatory failure. Eating was possible without swallowing the wrong way. Hematological disturbances corrected slowly and transfusions could be discontinued by the 11th day. Hemoglobin was still low at 8 g/gldl and platelet count was still low at 50,000/mm^3^. Two weeks later, the hematological parameters had reached normal values.

Our patient firmly denied any intentional overdose and her psychiatrist confirmed she had stable bipolar disorder. However, we failed to find any infectious or iatrogenic trigger which led to this extremely high lithium concentration. So we cannot formally exclude an intentional overdose. Acute kidney injury with oliguria may have increased the toxicity of lithium although the etiology of acute kidney failure remained unclear.

The lithium was definitely stopped and she received divalproate instead. As a new seizure happened, she was granted to an antiepileptic dose of divalproate. Polyuria and polydipsia remained. She still complained about sensory defect and thin motricity weakness.

To conclude, we report the case of a severe acute on chronic lithium intoxication with five scarcely reported complications.

## Discussion

Acute on chronic lithium poisoning is associated with a poorer prognosis as compared to non-previously exposed patients, mainly because of a more frequent central nervous system failure (CNS) [5]. In those cases, orotracheal intubation is required for 5% of the patients and 3% of them will present seizures [5]. Those patients do not show higher lithium level as compared to acute lithium poisoning. This may be linked to the slow diffusion of lithium which impedes it from penetrating the central nervous system in acute poisoning while chronically exposed patients have high lithium concentration in CNS before poisoning. As a matter of fact, lithium level does not seem to be the most important parameter to consider when treating lithium intoxication.

Lithium has already been tested for its erythropoietic side effect [6]. This effect might implicate glycogen synthase kinase 3 beta (GSKB3) inhibition, a repressive kinase which inhibits vascular endothelial growth factor synthesis. Patients under lithium therapy may show polynucleosis, as was noticed on our patient’s first biological exam. Effects on erythroid and megacaryocytic cells are less known, even though lithium has already been successfully used to cure aplastic anemias.

Dealing with lithium poisoning, cytopenias are scarcely reported. Lithium can reduce erythroid progenitor growth in vitro [7]. On mice [8], lithium treatment with 20 mg/kg daily can reduce erythropoiesis. To our knowledge, only one case was published concerning neutropenia [9]. We found one case of peripheral thrombocytopenia [10] and one case of severe thrombopenia with myocardial infarction during acute on chronic poisoning [11]. Only one case of pancytopenia during lithium therapy has been published [12]: the case reported is about a chronically treated woman who never got intoxicated. Her lithium level had always been below 0.76 mmol/l and she did not show any other sign of lithium toxicity.

The lithium poisoning may be responsible for the pancytopenia we observed. Hemoglobin content gradually fell and biological exam including bone marrow aspiration indicated central cytopenias. Other drugs were unlikely to be responsible for the pancytopenia we observed as clobazam and levetiracetam had been introduced less than 48 h before hemoglobin, platelets, and white blood cells began to fall. We investigated other possible factors which all turned back negative. Nonetheless, there are very few case reports of hematologic toxicity during lithium poisoning and there may be other causes we failed to find. In our defense, lithium levels are scarcely as high as the one reported in our case. Hematotoxicity might only occur for very elevated lithium levels, either blood level or total body level. When a cytopenia occurs, bone marrow aspiration should always be practiced as some cytopenia may be provoked by immunologic way as shown in [10], a situation that may benefit from corticoids.

Polyneuropathy is a very rare concern during lithium poisoning but we were doubtful about it being caused by critical illness polyneuropathy with the precocity of occurrence, the lack of main risk factors of reanimation polyneuropathy (no use of curare or corticosteroid), and the impairment of cranial nerve function. Main alternative diagnoses were also ruled out: there was no dyscalcemia or thyroid dysfunction; infectious and immunologic disorders had previously been investigated. We choose not to repeat the lumbar puncture because of the non-demyelinating character of the neuropathy.

We found only few cases of such a condition [13–15]. Pathophysiology is still controversial and may implicate lithium accumulation in the neural cells. Most of the cases concern acute on chronic poisoning [13–15] with lithium levels mildly increased, around 3.2 mmol/l at presentation. Polyneuropathy often begins 3 or 4 days after the ICU admission, sometimes when lithium level has decreased below the toxic scale [15]. Most authors report long tract neuropathy and oculomotor abnormalities [13–15]. There must be no sign of demyelinating neuropathy. Diabetes insipidus is a common finding on these patients, and it may be either a risk factor for developing polyneuropathy or a sign of acute on chronic intoxication. Polyneuropathy decreases most of the times and major sequels are very scarce though it may take several months before patients could be able to walk [15].

The case we report is similar to the previously published cases concerning the delayed symptoms and the spontaneous recovery. As in the case published by Chan [15], polyneuropathy was associated with the new onset of diabetes insipidus. However, our patient would not experience complete recovery of her motricity. She would still present impaired thin motricity and paresthesia. This may be linked to the longer exposure to lithium as compared with the cases published [13–15], or the severity of the lithium intoxication. We cannot exclude a belated recovery we are too early to find. In our case report, polyneuropathy lengthened mechanical ventilation period by at least 4 days. As a matter of fact, it seems important to wait a few days before proceeding to tracheotomy when a lithium-intoxicated patient shows early polyneuropathy that impedes extubation from being proceeded.

One should consider extra renal replacement therapy if lithium level is above 4 mmol/l [2] with impaired kidney function or whenever there is neurological symptoms as lithium levels barely correlate with neurological involvement. Above 5 mmol/l [2], continuous renal replacement therapy (CRRT) is required whatever the kidney function. One should prefer hemodialysis over hemofiltration due to the ionic diffusive nature of lithium. The only extrarenal epuration technic available in our center at the admission was CVVHDF, the reason why our patient did not benefit from hemodialysis. Even though all the described complications occurred after the beginning of CVVHDF, we do not think that CVVHDFH was ineffective. Complications must have occurred because organ injuries had already been settled but were still infraclinic when we admitted this patient in our ICU. For instance, pancytopenia was the first manifestation that occurred and the last one is alopecia. It is very close to what is described when using cytotoxin: quick-regenerating tissues like bone marrow are the first ones to suffer from the intoxication and slow-regenerating ones like hair are the last ones. In our point of view, the success of lithium poisoning treatment must focus on the control of seizure and status epileptius rather than belated toxicities. As status epilepticus was rapidly controlled, we decided to pursue CVVHDF; we would have changed our CRRT technic if it had not improved.

Due to its ionic properties, there is an important intracellular pool of lithium, and a major risk of “rebound” after a CRRT session. We choose to determine lithemia before and after each CVVHDF session and we did not find any rebound. However, it is very unlikely we missed any important rebound because our patients kept severe kidney injury and could not lower her lithium concentration by any way when CVVHDF was discontinued. We think that whether a rebound had occurred after CVVHDF, lithium concentration would have remained high until the next CVVHDF session because the residual clearance of lithium was drastically reduced. Data are missing concerning the effects of early CRRT to prevent intoxication sequels, namely the underestimated ones like peripheral neuropathies and diabetes insipidus.

Concerning kidney function, chronic exposure to lithium does not seem to affect the glomerular filtration rate [16]. The urine maximal concentration ability is though significantly impaired [2]. Polyuria is actually a very classic chronic lithium exposure complication. Acute poisoning can increase the risk of a polyuria to happen [17]. Lithium intoxication is often associated with new onset of diabetes insipidus nephrogenicus. This situation can lead to dehydration and consequently increase lithium retention. For instance, patients presenting with diabetes insipidus nephrogenic could show a 25-fold increased risk of developing CNS involvement during lithium poisoning [18]. When lithium cannot be stopped, amiloride may be used to lower the risk of the patient developing diabetes insipidus [19]. This effect on urine concentration may be mediated by adenylate cyclase [20] inhibition that impedes aquaporins from migrating to the renal collecting duct. GSk3B is suspected to participate to this diabetes insipidus but its full action is still controversial.

Lithium poisoning may cause alopecia. Most of cases are case report [21] and a recent meta-analysis has not shown any increased incidence of alopecia concerning chronically treated patients. However, this review does not focus on lithium poisoning. Alopecia may be linked to GSk3B inhibition as this kinase promotes hair growth. In spite of this complication not being a life-threatening one, alopecia may defavorably impact the patient’s quality of life.

## Conclusion

To conclude, we report the case of a severe lithium poisoning requiring urgent CVVHDF. We noticed hematological, neural, and nephrologic complications which are scarcely described. The patient was cured but kept unattended sequels, among which are distal neuropathy and diabetes insipidus.

## Abbreviations

ALAT: Alanine-amino transferase
ASAT: Aspartate-amino-transferase
CNS: Central nervous system
CRRT: Continuous renal replacement therapy
CVVHDF: Continuous veno-venous hemodiafiltration
GSK3B: Glycogen synthase kinase 3 beta
HIV: Human immunodeficiency virus
ICU: Intensive care unit
